# Thallium Exposure Secondary to Commercial Kale Chip Consumption: California Case Highlights Opportunities for Improved Surveillance and Toxicological Understanding

**DOI:** 10.3390/ijerph22081235

**Published:** 2025-08-07

**Authors:** Asha Choudhury, Jefferson Fowles, Russell Bartlett, Mark D. Miller, Timur Durrani, Robert Harrison, Tracy Barreau

**Affiliations:** 1 California Department of Public Health, Environmental Health Investigations Branch, Richmond, CA 94804, USAtracy.barreau@cdph.ca.gov (T.B.); 2 California Department of Public Health, Occupational Health Branch, Richmond, CA 94804, USA; 3 Epidemic Intelligence Service, Centers for Disease Control and Prevention, Atlanta, GA 30329, USA; 4 Western States Pediatric Environmental Health Specialty Unit, San Francisco, CA 94143, USA; mark.miller2@ucsf.edu (M.D.M.); timur.durrani@ucsf.edu (T.D.); 5 Division of Occupational Environmental and Climate Medicine, University of California San Francisco, San Francisco, CA 94143, USA; 6 California Poison Control System, San Francisco Division, San Francisco, CA 94143, USA

**Keywords:** thallium, food safety, emerging contaminant, human health

## Abstract

Background: Thallium is a metal that is ubiquitous in our natural environment. Despite its potential for high toxicity, thallium is understudied and not regulated in food. The California Department of Public Health was alerted to a household cluster of elevated urine thallium levels noted among a mother (peak 5.6 µg/g creatinine; adult reference: ≤0.4 µg/g creatinine) and her three young children (peak 10.5 µg/g creatinine; child reference: ≤0.8 µg/g creatinine). Objectives: This case report identifies questions raised after a public health investigation linked a household’s thallium exposure to a commercially available food product. We provide an overview of the public health investigation. We then explore concerns, such as gaps in toxicological data and limited surveillance of thallium in the food supply, which make management of individual and population exposure risks challenging. Methods: We highlight findings from a cross-agency investigation, including a household exposure survey, sampling of possible environmental and dietary exposures (ICP-MS analysis measured thallium in kale chips at 1.98 mg/kg and 2.15 mg/kg), and monitoring of symptoms and urine thallium levels after the source was removed. We use regulatory and research findings to describe the challenges and opportunities in characterizing the scale of thallium in our food supply and effects of dietary exposures on health. Discussion: Thallium can bioaccumulate in our food system, particularly in brassica vegetables like kale. Thallium concentration in foods can also be affected by manufacturing processes, such as dehydration. We have limited surveillance data nationally regarding this metal in our food supply. Dietary reviews internationally show increased thallium intake in toddlers. Limited information is available about low-dose or chronic exposures, particularly among children, although emerging evidence shows that there might be risks associated at lower levels than previously thought. Improved toxicological studies are needed to guide reference doses and food safety standards. Promising action towards enhanced monitoring of thallium is being pursued by food safety agencies internationally, and research is underway to deepen our understanding of thallium toxicity.

## 1. Introduction

Thallium is a metal with high potential for toxicity, based on dose, which can result in death or multiorgan involvement. Thallium was used historically in rodenticides, which were banned in the United States in 1975 after continued accidental thallium exposures. Thallium’s tasteless, colorless, and odorless nature has likely contributed to its use in intentional, fatal poisonings and its moniker “the poisoner’s poison” [1]. Although production of thallium-containing products in the United States has largely ceased [2], thallium is naturally occurring in trace elements in the Earth’s crust. Human exposure can be amplified through anthropogenic activity such as mining, which mobilizes geologic thallium deposits; industrial processes such as those at coal-burning plants, which release emissions; and uptake into crops, which can introduce thallium in the diet [3,4,5].

Thallium exposure can occur through inhalation, ingestion, and through the skin. However, acute exposures can be misdiagnosed, because exposed patients present with nonspecific signs and symptoms, including pain in extremities, gastrointestinal irregularities, abdominal pain, and rashes [6]. Hair loss is a hallmark feature that occurs several days after exposure. Many patients have long-term neurologic sequelae even with treatment and rehabilitation [6]. During 2018–2022, the American Association of Poison Control Centers reported 127 cases of thallium poisoning in persons aged ≥20 years. Cases among young children are more rarely reported, with just eight cases noted among persons aged ≤5 years during 2018–2022 [7,8,9,10,11].

Despite its high toxicity, thallium is relatively understudied and is not regulated in food. There is little known on the toxicological effects of low-dose or chronic exposures on sensitive subpopulations, including children, and a lack of surveillance exists regarding environmental exposures, including in the food supply. Regulatory efforts to manage thallium exposures are limited to the development of a drinking water standard in 1994. In 2009, the U.S. Environmental Protection Agency (EPA) rescinded a previous thallium reference dose, given lack of confidence in a key animal study guiding its development. Recent regulatory efforts to develop heavy metal reference levels in food, such as the U.S. Food and Drug Administration (FDA) Closer to Zero campaign focusing on baby food started in 2021, have not included thallium.

## 2. Objectives

We investigated a thallium exposure in a family, including three young children, and identified a commercially available food product as the source. We provide an overview of the public health investigation that demonstrates the extensive sampling, testing, and investigative effort required to identify possible sources of thallium exposure. We then explore concerns, including gaps in toxicological data and limited surveillance data regarding thallium in the food supply that make management of exposure risks at both individual and population levels challenging for public health officials.

## 3. Household Thallium Investigation

### 3.1. A Household Cluster of Elevated Random Urine Thallium Levels

In August 2022, the Western States Pediatric Environmental Health Specialty Unit (PEHSU) was contacted by a family with elevated urine thallium levels in four of five members of a family living in a semirural, agricultural setting. PEHSU alerted the California Department of Public Health (CDPH) to the cluster and requested assistance in investigating the source. Medical history and laboratory results were provided by the family and their physicians and verified by CDPH.

**Figure 1:**
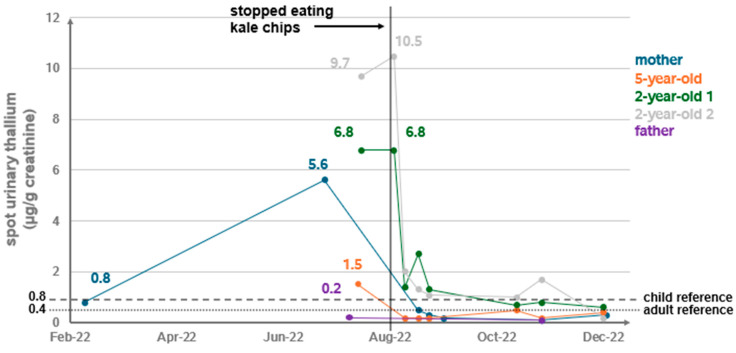
Random urine thallium concentrations of family members.

The mother was aged 39 years and had a history of well-controlled hypothyroidism. She was tested for metals in February 2022 after reporting months of nonspecific symptoms including numbness and tingling in hands and feet, loose bowel movements, hair loss, and fatigue, along with episodic night sweats and dizziness. The thallium concentration in this spot urine sample was 0.8 µg/g creatinine (Figure 1), which is elevated compared with the adult reference range (≤0.4 µg/g creatinine) provided in the clinical laboratory results. The upper range of normal on the laboratory test is consistent with the 95th percentile from our most recent National Health and Nutrition Examination Survey data (0.456 µg/g creatinine for persons aged ≥20 years in 2017–2018) [12]. Upon retest in early July 2022, the mother’s spot urine thallium concentration had risen to 5.6 µg/g creatinine, prompting testing of the remaining family members. In July, the mother also underwent nerve testing (sensory and motor conduction tests, and needle electromyography) in arms and legs. The sensory conduction tests showed evidence of mild axonal sural neuropathy bilaterally.

Urinary thallium concentrations for her child aged 5 years and twins aged 2 years were also elevated when compared with NHANES data (1.5, 6.8, and 9.7 µg/g creatinine, respectively, compared with a 95th percentile level of 0.793 µg/g creatinine for children aged 3–5 years in NHANES, 2017–2018). The father’s spot urine thallium levels, when tested in March 2022 and July 2022, were found to be within the adult reference range.

Medical history of the twins was notable for frequent upper respiratory infections. Both twins began experiencing loose stools in February 2022. Additionally, in February, twin 1 began experiencing episodes of abnormal eye movements, altered attention, and short self-resolving episodes of leg pain that limited walking. In March 2022, twin 2 was noted by parents to have shaking in the arm when feeding himself, and occasional whole-body jerks that appeared involuntary. Twin 1 also started excessively sweating in August. In August, both twins were evaluated by ophthalmology. Twin 1 was found to have bilateral optic nerve drusen. Twin 1 underwent MRI, which was normal. Both twins underwent electroencephalogram (EEG) during awake and sleep states, one lasting 24 h and the other 30 min, which were also normal and without correlates for seizure. The symptoms involving the extremities persisted. Assessment by a neurologist noted mildly increased tone of ankles bilaterally of twin 1 and otherwise did not yield definitive diagnoses. The older child and father did not experience unusual symptoms during this period. Poison Control was consulted and determined that levels and symptoms did not merit chelation.

### 3.2. Public Health Investigation and Findings

Our public health investigation included a household exposure survey, environmental assessment, and clinical monitoring. This activity was reviewed by the Centers for Disease Control and Prevention (CDC) and California Health and Human Services Agency Committee for Protection of Human Subjects, deemed not research, and was conducted consistent with applicable federal law and CDC policy (45 C.F.R. part 46, 21 C.F.R. part 56; 42 U.S.C. Sect. 241(d); 5 U.S.C. Sect. 552a; 44 U.S.C. Sect. 3501 et seq). The Federal Bureau of Investigation and Department of Homeland Security were also involved in the investigation and determined no criminal intent.

#### 3.2.1. Household Exposure Survey and Environmental Assessment

We conducted a detailed exposure survey to collect information about the environment in and around the home, occupational risks for the parents, and the family’s diet and use of supplements and cosmetic products. Possible household sources identified through the survey included (1) a well on the property used by the family for daily bathing, (2) imported bottled water consumed by the family, (3) consumption of brassicas and cruciferous vegetables known to accumulate thallium when grown in contaminated soil, (4) soil deposits exposed when yard is regularly turned over, or (5) pesticides or rodenticides remaining in the older (pre-1970s) home, adjacent outdoor structure, or grounds. The family was advised to drink bottled water until further notice and cease consumption of food products such as kale chips that might have elevated thallium levels. The children and a parent temporarily relocated out of the home.

CDPH worked with a U.S. Environmental Protection Agency (EPA) On-Scene Coordinator to collect 25 samples, including surface soil, drinking water, indoor air, and surface dust (wipe) samples from the property. Samples were analyzed using inductively coupled plasma mass spectrometry (ICP-MS) for thallium and other metals. None of the environmental samples contained thallium at detectable levels (Table 1). Other metals were found at levels well below amounts known to cause harm.

The only concerning survey item reported by the family was that the mother and children regularly consumed kale chips. The mother and 5-year-old had started eating this snack in mid-2021, and the 2-year-olds began eating the chips in early 2022. Consumption was intermittent and variable, with peak consumption estimated as approximately 1–3 28 g servings daily for several days in a row. The father did not eat kale chips.

We purchased kale chips of the same brand consumed by the family and tested them for thallium first with a handheld S1 TITAN X-ray fluorescence spectrometer (XRF) (Bruker, Karlsruhe, Germany) and then with ICP-MS. Whereas the XRF did not detect thallium above the device detection limit of 5 mg/kg, ICP-MS analysis measured thallium in the kale chips at 1.98 mg/kg and 2.15 mg/kg. We shared the findings of our investigation with FDA, which alerted the United States-based manufacturer. Beyond this, CDPH is unaware of any investigations into the source of the thallium in the kale chips, preventive controls taken by the company to monitor or minimize thallium in the product, or any actions to alert consumers.

#### 3.2.2. Clinical Intervention and Ongoing Monitoring

After the family ceased consumption of the kale chips, subsequent urinary thallium measurements of the family showed declining concentrations. The family moved back into their home after nearly two months away. We continued to monitor the family’s clinical symptoms and urinary thallium measurements for approximately 1 year. Certain signs and symptoms experienced by the family abated after removal of kale chips from the diet, whereas others persisted. By August 2023, the mother’s gastrointestinal symptoms had resolved, and her sensory neuropathy improved subjectively. Repeat nerve conduction studies in June 2023 showed objective improvement in sural sensory responses to normal range. Hair thinning continued to be a concern. Twin 1 continued to have ongoing loose bowel movements. His alertness improved, but he continued to have periods of agitation associated with leg pain and difficulty walking. Twin 2 had normalization of bowel movements and improvement in arm shaking when feeding. Both twins worked on expressive language skills with speech therapy services, which were terminated by their third birthday without concern. Their pediatrician found that they were meeting developmental milestones.

## 4. Discussion

This investigation of a household cluster of elevated urine thallium concentrations, above national 95th percentiles, pointed toward the family’s consumption of contaminated commercially available kale chips as the likely cause. Elevated thallium levels were not observed in the family member not consuming the kale chips, and levels dropped in affected family members when consumption ceased. Serial creatinine-corrected spot urine testing allowed us to detect changes from the initial values reported to CDPH. Given the short half-life of thallium in blood, urine testing is preferred to measure chronic exposure. As children have lower muscle mass and excrete less creatinine than adults, their creatinine-corrected urine levels should only be compared to reference values derived from children from similar ages, as opposed to adults [14]. The biological half-life of thallium in urine is estimated to range from 10 to 30 days [15], which can explain the persistent elevation in urine samples a few days after the family stopped eating the kale chips. Urine of the twins in diapers was not collected in sealed collection cups; however, the survey and environmental review of the home did not identify any external sources of thallium that might have caused contamination of the samples.

Many questions arose as a result of this investigation. A dearth of research on lower-dose thallium exposure and the lack of a reference dose complicated our ability to contextualize our case findings. Although exposure is evident through the elevated urinary thallium levels, definitively determining if these levels were associated with the symptoms experienced is difficult. The mother’s signs and symptoms included peripheral neuropathy, gastrointestinal irritation, and hair loss; however, this constellation of symptoms has generally been noted in case reports of acute thallium toxicity involving substantially higher doses. Two very limited studies of low-dose chronic thallium exposure included subjects with self-reported neurologic symptoms at similar urine levels as the mother’s, whose neuropathy did improve with falling urine levels [16,17]. The 2-year-old twins also had complex symptoms that may be secondary to a litany of medical etiologies; yet, extensive medical testing by the family pediatrician and other specialists, including in neurology, endocrinology, and ophthalmology, did not identify a cause. Given the unique vulnerability of children to neurotoxicants, we sought to understand how thallium may have accumulated in this particular product. Limited testing of food products hampers our understanding of how pervasive thallium might be in our food supply and population risk for dietary exposure. The lack of a reference dose further complicates the ability to set regulatory limits for thallium in food. We summarize relevant research and regulatory context here and opportunities to further our understanding.

### 4.1. Opportunities for Improved Toxicological Understanding

#### 4.1.1. Lack of Human Studies Representing Chronic Thallium Exposure

We first looked to data regarding humans to understand possible health implications associated with the urine thallium levels noted in our investigation. We found that data on human thallium toxicity is primarily from case reports describing acute poisonings often at high doses and corresponding substantially elevated serum levels [15,18,19]. Gastroenteritis, polyneuropathy, and alopecia are regarded as the classic clinical triad of thallium poisoning. However, toxicity can occur in a range of organs, including the liver, nervous system, kidney, cardiovascular system, skin, and reproductive system [18], and can even cause death. In contrast, substantially fewer data on chronic, low-dose exposures are available. Chronic toxicity is described across reviews and textbooks as having more mild and variable symptomology, but is rarely presented alongside exposure data [15,18]. Two studies describing low-dose exposure were considered most instructive for a 1996 review of the toxicological data by the International Programme on Chemical Safety (IPCS), a joint taskforce including the World Health Organization [15]. These included a population study that surveyed a German community near a cement plant where thallium emissions were discharged to the air, deposited in soil, and then taken up by crops being consumed locally [16]. Positive correlations were observed between thallium levels in urine (mean: 5.2 µg/L) and hair and subjective self-reported symptoms of sleep disorders, tiredness, weakness, nervousness, headache, psychological alterations, and some neurologic and muscular symptoms such as paresthesias and pains in muscles and joints. However, long-term health outcomes were not assessed [16]. The second study focused on occupational exposures to 36 cement plant workers. Thallium in blood, urine, and hair was measured along with neurological assessments, including nerve conduction and EEG. Approximately one-third of the total workers were noted to have a sign or symptom of neurologic disorder, such as paresthesia, numbness of toes and fingers, “burning feet”, and muscle cramps. Eight of the patients had clinical evidence of sensory distal polyneuropathy; motor and sensory nerve conduction were normal, but sural nerve conduction velocity was reduced in seven. Five workers had elevated urinary thallium levels, defined by the paper as >5 µg/L. Because half the workers had comorbid conditions (e.g., diabetes) that might also present with neurologic symptoms, no conclusions could be made about the levels of thallium found in the laboratory and neurologic parameters assessed [17]. Upon review, IPCS declined to create a health-based exposure limit pending improved dose–response studies. However, in considering the two population studies above, they offered that detrimental health effects are not expected at urine thallium concentrations below 5 µg/L in urine (or 6.4 µg/g creatinine), corresponding with a daily oral intake of approximately 10 µg/day [15]. Above these levels, the magnitude of the risk and severity of adverse effects is uncertain [15].

Although this benchmark has been useful to contextualize the laboratory findings in our investigation, we can appreciate the dearth of human data and considerable uncertainty in generating the 1996 IPCS threshold. In recent years, additional studies have examined effects of thallium exposure at substantially lower doses. Particularly vulnerable subgroups include children and pregnant women [20]. One case series followed six children aged 1–9 years with relatively low urine thallium concentrations (13.4 µg/L to 60.1 µg/L) over a four-year period [21]. Four experienced slightly poor appetite, but none described showed classic signs or symptoms of thallium poisoning. Despite treatment and reduction in urinary thallium levels below the threshold of detection, all children had mild but persistent abnormal laboratory results that might suggest some effect on liver or kidney function, changes in lipid metabolism, or myocardial damage [21]. In another study, women with urine thallium levels of >0.8 µg/g creatinine (or 0.47 µg/L unadjusted) were shown to have greater risk for preterm birth, compared with women with <0.36 µg/g creatinine [22]. Low-level maternal thallium exposure during pregnancy, with similar corresponding urine thallium levels, has also been associated with increased risk for low birth weight [23,24] and alterations in mitochondrial DNA in newborns [25,26]. A prospective cohort study of coal processing plant workers found that those with urine thallium levels >0.86 µg/L had larger declines in lung function (measured by FEV_1_), compared with those with urine thallium levels ≤0.86 µg/L [27]. Decreases in certain thyroid hormones have been found to be associated with increases in urine thallium levels in 2007–2008 NHANES data, where the maximum urinary thallium levels was 0.21 µg/L [28]. These studies, of both low-dose occupational and community exposures, demonstrate possible multiorgan and other widespread effects. However, studies of low-dose thallium exposure are relatively few and limited in the information they can provide. This is because they often used cross-sectional design or otherwise limited follow-up duration, and rare inclusion of children, a possible vulnerable subpopulation to this known neurotoxicant [29].

#### 4.1.2. Role for Further Animal Studies

Given the limitations in observational human studies, animal studies might provide opportunities to explore thallium toxicity further. Toxicological data are necessary to guide the development of safe levels for individual consumption and determine actions levels at which products are taken off shelves. At present, animal studies on thallium toxicity are also limited. In 2009, EPA reviewed toxicology literature for thallium for the purpose of developing a reference dose (RfD) [18]. EPA’s reference doses are intended to provide estimates of a daily oral exposure to the human population that is likely to be without an appreciable risk for deleterious effects during a lifetime [30]. The previously published RfD (8.0 × 10^−5^ mg/kg/day to 9.0 × 10^−5^ mg/kg/day) for soluble thallium salts [18] had been based on the findings of a single unpublished study, a 90-day oral drinking water study in Sprague-Dawley rats [31] (Appendix A: Table A1). On re-review, EPA withdrew this RfD because of a lack of confidence in the principal study, including debates about dose selection and how the endpoint, alopecia, was evaluated [18]. EPA determined that clear evidence existed of dose-dependent effects in different systems. However, the evaluated studies were deemed to be too poor in quality to guide a reference dose and our understanding of neurotoxicity, developmental and reproductive toxicity, and carcinogenicity [18].

Another complication is the lack of an accepted and understood mode of action for thallium toxicity [32,33] and a toxicokinetic model that might help determine how and where thallium is distributed in key organs and tissues. A hypothesis has been proposed that thallium competes with potassium in its mode of action [15,33]. However, this theory has not been sufficiently explored to facilitate examination of crucial early biochemical effects that might lead to an identifiable set of endpoint pathologies.

Recognizing these gaps in the understanding of thallium toxicity, in December 2022, we nominated thallium to the National Institutes of Health National Toxicology Program (NTP) for further study regarding developmental neurotoxicity and chronic effects, including carcinogenicity and reproductive toxicity. This builds on the EPA’s nomination of thallium in 2015 for review of additional neuro- and repro-toxicity endpoints [34]. NTP has initiated multiple major toxicology studies on thallium compounds, including two 90-day studies in rats and mice, a 14-day range finding study in mice, in vitro genotoxicity studies, and a special cardiovascular toxicity study in rats [35]. Results from a developmental toxicity study were released in May 2023. The study of short-term toxicity of thallium salts in drinking water on rats and mice was intended to mimic potential human exposure to thallium in contaminated drinking water and evaluate potential hazards during pregnancy and development [36]. Lowest observed effect levels of 12.5 mg/L (rats) and 25 mg/L (mice) were associated with increased alopecia in rat pups and significantly decreased body weights for rats and mice. The no observed adverse effect levels were determined to be 6.25 mg/L (rats) and 12.5 mg/L (mice), equivalent to doses of 0.8 mg/kg bw/day (rats) and 2.0–2.2 mg/kg bw/day (mice). Measurements of thallium in maternal plasma and amniotic fluid, fetuses, and pup plasma demonstrated maternal transfer of thallium to offspring during gestation and lactation [36].

Further animal studies on the subchronic and chronic effects of thallium toxicity should provide additional dose–response data for the development of a reference dose and health-based exposure guidance, although it might be several years until sufficient data have been amassed and reviewed by regulatory agencies. In the interim, opportunities for improved food safety surveillance and regulation might exist.

### 4.2. Opportunities for Improved Food Safety Surveillance and Regulatory Actions

#### 4.2.1. Thallium Accumulation in Brassicas

Because of the levels of thallium observed in the kale chips we tested, we sought to determine how pervasive thallium might be in our food supply and how it might have accumulated in our sample of this product.

Vegetables have been recognized as a possible source of dietary thallium exposure since the 1960s [15]. Recent dietary surveys testing foods in the United Kingdom, New Zealand, and Italy have shown that brassicas, in particular, have some of the highest concentrations of thallium (mean concentrations ~5 µg/kg; maximum 20 µg/kg) [37,38,39]. Kale and other members of the Brassicaceae family of plants, which includes cabbage, broccoli, and Brussels sprouts, can bioaccumulate thallium into the leaves of the plant up to 80 times the concentration of the soil [5,40,41]. Brassicas grown in thallium-rich soils (1.5–124 mg/kg) have been seen to reach levels of 110–495 mg/kg [42,43], considerably higher than the thallium content (2 mg/kg) detected in the commercial kale chips consumed by the family in our investigation. Topsoil from across the contiguous United States has been found to have a mean concentration of 0.4 mg/kg thallium, but levels vary widely (range: <0.1–8.8 mg/kg) [13]. Further enhancement of thallium levels in kale chips could result by introduction or magnification during manufacturing processes, such as dehydration. For example, in the 2016 New Zealand Total Diet Study, potato crisps were noted to have on average 10 times more thallium than peeled potatoes (mean concentrations: 0.464 mg/kg vs. 0.0048 mg/kg) [37]. Intrinsic or manufacturing processes that contribute to accumulation of thallium in food products might also contribute to accumulation of other metals; for example, brassicas have been observed to uptake lead, cadmium, and other metals, and have even been studied as phytoremediators for contaminated areas [44].

#### 4.2.2. Role for Further Monitoring of Thallium in Food Supply

Although multiple ways exist by which thallium can accumulate in brassica vegetables, we do not know the scale at which thallium is present in the United States food supply. Without further surveillance, we lack the ability to assess population risk of contamination.

At the time of our investigation, thallium testing was not included in the United States Total Diet Study, which FDA uses to monitor levels of nutrients and contaminants in foods and to estimate daily dietary intake of U.S. consumers. As previously discussed, we also lack a national health-based reference dose to guide understanding of safe levels of thallium exposure in food and soil. In the absence of these data, dietary surveys conducted worldwide have sought to compare estimated dietary intake of thallium with health-based thresholds derived from the 10 µg/day oral intake threshold identified by IPCS. Studies in the United Kingdom, Canada, and New Zealand have found estimated dietary intakes of thallium highest among toddlers, with intakes that exceed the derived health-based thresholds [37,45,46]. Consumption of a single, 28 g serving/day of kale chips with concentration of ~2 mg/kg thallium, as noted in our investigation, would also result in intake levels that exceed both the historic EPA reference dose [18] and IPCS threshold [15].

In April 2023, at the 16th annual Codex Committee on Contaminants in Foods, the United States proposed adding thallium to a priority list of contaminants for a full evaluation (toxicological and exposure assessment) by the Joint Food and Agricultural Organization of the United Nations and WHO Expert Committee on Food Additives. The United States delegation stressed that thallium is an acute and chronic toxicant in need of an international health-based guidance value and quantifying its presence in the food supply [47]. They proposed adding thallium into the Global Environment Monitoring System/Food Contamination Monitoring and Assessment Programme to facilitate data collection internationally on thallium levels in food [47,48]. The United States delegation also reported at that meeting that FDA is planning analyses for thallium in brassica-containing foods, in baby foods, and in United States Total Diet Study collections [47,49]. In August 2023, a preliminary FDA analysis of brassica-containing products found that kale chips had the highest levels of thallium. The highest concentration of thallium was 2.41 mg/kg [50], greater than the samples tested in our investigation. Notably, the most recent 2024 New Zealand Total Diet Study focused on infants and young children, recognizing them as particularly vulnerable to dietary contaminants given the large amount of food consumed relative to their small body weight. Results from their survey conducted in mid-2024 noted fresh kale (kale chips not tested) had the highest thallium content of any food measured, as high as 0.29 mg/kg [51].

FDA has since added thallium to a list of chemicals in the food supply under review [52]. Until further toxicological research is conducted, developing action levels that determine when products should be taken off the shelves will be challenging. This leaves the door open for ongoing exposures and health consequences, particularly among vulnerable populations. Additionally, without action levels, manufacturers are largely responsible for ensuring their product is safe for consumption. Data from FDA’s preliminary analysis on levels of thallium in foods could be useful for manufacturers in guiding product and soil testing. Additionally, manufacturers of products identified to have higher levels of heavy metals, such as arsenic, lead, cadmium, and mercury, recognized by federal agencies and in state regulations as possible environmental contaminants [53], could consider expanding testing to include monitoring of thallium, given the potential for concomitant contamination.

## 5. Conclusions

Our investigation of a cluster of elevated spot urine levels and reported associated symptoms among a family showed commercially available kale chip consumption as a potential source. The elevated urine thallium levels, discovered after the mother reported persistent peripheral neuropathy, gastrointestinal symptoms, and hair loss, lessened after cessation of kale chip consumption. Thallium can bioaccumulate in kale and other brassica vegetables, with levels affected by thallium concentration of the soil in which the crop is grown. Dietary intake studies around the world have estimated thallium intake by toddlers to exceed health-based guidance values derived from the IPCS oral intake threshold. We largely lack human data to understand implications of low-dose, chronic exposures of this known neurotoxicant, particularly among children. Improved understanding of the toxicology of thallium might guide a national oral reference dose and food standards such as action levels. Furthermore, enhanced monitoring is key to understanding the scale of the problem in our food supply. Progress toward these goals is underway by public health and research agencies, although many opportunities exist for further action.

## Figures and Tables

**Table 1:** Summary of thallium (Tl) levels found in environmental samples.

Sample Media	Number of Samples Collected	Range of Tl Found	Summary of Sample Locations
Surface soil *	6	ND (<0.11)–0.16 mg/kg	Children’s play area and various backyard locations, and family’s vacuum cleaner.
Drinking water	4	ND (<0.001) mg/L	Family’s private well and purchased water the family uses for drinking.
Indoor air	7	ND (<0.009) µg/m^3^	Crawl space, attic, children’s bedrooms, living room, and kitchen.
Surface dust (wipe) **	8	ND (<0.013) mg/sample	HVAC filter, used mobile air filter, the children’s slide in the backyard play area, and floor vents in all bedrooms and living room.

Note: ND = not detected above the laboratory method of detection limit; mg/kg = milligrams per kilogram; mg/L = milligrams per liter; µg/m^3^ = micrograms per cubic meter; mg/sample = milligrams per sample collected. * Reported US Geological Survey background thallium level (mean) 0.4 mg/kg [13]. ** Thallium laboratory reporting limit dependent on quantity of dust obtained from wipe sample.

## Data Availability

The original contributions presented in this study are included in the article. Further inquiries can be directed to the corresponding author.

## Abbreviations

The following abbreviations are used in this manuscript:

CDPH	California Department of Public Health
NHANES	National Health and Nutrition Examination Survey
EPA	United Stated Environmental Protection Agency
FDA	United States Food and Drug Agency
IPCS	International Programme on Chemical Safety
RfD	Reference dose
NTP	National Institutes of Health National Toxicology Program

## Appendix A

**Table A1:** Summary of reference exposure levels or urine thallium thresholds and supporting studies.

Organization	Reference Levels (mg/kg-day) or Urine Tl (µg/L)	Supporting Studies	Limitations
U.S. Environmental Protection Agency (EPA)	RfD range: 8.0 × 10^−5^ to 9.0 × 10^−5^ (withdrawn in 2009)	[18] U.S. EPA. IRIS Toxicological Review of Thallium And Compounds. U.S. Environmental Protection Agency. September 2009.EPA/635/R-08/001F. [31] Stoltz ML, Stedham MA, Brown LK, et al. 1986, Subchronic (90 day) toxicity of thallium (I) sulfate in Sprague-Dawley rats. Report to U.S. EPA, Office of Solid Waste, Washington, DC, by Midwest Research Institute, Kansas City, MO.	Lack of confidence in the key toxicological study in rats (Stoltz 1986; as cited by EPA 2009). The data gaps of the 90-day oral gavage study discussed by EPA in their review included: “High background incidence of alopecia, lack of histopathological examination of skin tissue in low- and mid-dose groups, and inadequate examination of objective measures of neurotoxicity”.
U.S. EPA Superfund Risk Assessment	PPRTV: subchronic and chronic provisional RfDs ranging from 1 × 10^−5^ to 4 × 10^−5^	[54] U.S. EPA. Provisional Peer-Reviewed Toxicity Values for Thallium (Soluble Salts). U.S. EPA, Washington, DC, EPA/690/R-12/026F, 2012.	Used data from 1986 study by Midwest Research Institute.
International Programme on Chemical Safety (IPCS) proposed in 1996 Environmental Health Criteria (EHC) 182	EHC 182 aligned with the conclusion of the 2009 U.S. EPA review that no health-based guidance value could be determined. IPCS proposed to base a safe intake of thallium on the 5 µg/L urine value, which was reported to be roughly equivalent to a daily intake of 10 µg/day.	[15] International Programme on Chemical Safety. Environmental Health Criteria 182 Thallium. World Health Organization. 1996.	Based on two epidemiological studies involving thallium-exposed workers and persons living near an industrial source. No developmental neurotoxicity studies that could serve to guide a health protective risk assessment threshold for thallium in pregnant women, adults of reproductive age, or young children.
